# An Analysis of Repeated High Intensity Efforts (RHIE) across Different Competition Levels in Elite Rugby Union

**DOI:** 10.3390/sports10100151

**Published:** 2022-10-11

**Authors:** Adam Sheehan, Shane Malone, Anthony Weldon, Aled Waters, Kieran Collins

**Affiliations:** 1 Munster Rugby, High Performance Unit, University of Limerick, Castletroy, V94 X9VK Limerick, Ireland; 2 Human Performance Lab, Faculty of Science, School of Biological Health and Sports Sciences, Technological University Dublin, Tallaght, D24 AC60 Dublin, Ireland; 3 The Tom Reilly Building, Research Institute for Sport and Exercise Sciences, Liverpool John Moores University, Liverpool L3 3AF, UK; 4 Department of Sports and Recreation, The Technological and Higher Education Institute of Hong Kong, Hong Kong; 5 Leicester Tigers, Mattioli Woods Welford Road Stadium, Aylestone Road, Leicester LE2 7TR, UK

**Keywords:** team sports, GPS, repeated efforts, collisions, physical demands

## Abstract

The current investigation aimed to understand the differing positional demands across two elite rugby union competitions, with special reference to high-intensity effort (HIE) and repeated high-intensity effort (RHIE) activity. Four hundred and forty-one (*n* = 441) individual game files from thirty-five competitive games from the European Rugby Champions Cup (tier 1; *n* = 8) and PRO12 League (tier 2; *n* = 24) were analysed. Players’ locomotor profiles were recorded using wearable global positioning system microtechnology (10 Hz Catapult S5, Catapult Innovations, Australia). Locomotor activities were classified as running (≥4.4 m∙s^−1^), high-speed running (≥5.5 m∙s^−1^), accelerations (≥2 m∙s^−2^) and decelerations (≤−2 m∙s^−2^). Data was gathered on collisions (≥4 g^−1^), high-intensity efforts (HIE), repeated high-intensity efforts (RHIE), average number of efforts within a RHIE bout (*n*) and maximal number of efforts within a RHIE bout (*n*). Overall locomotor differences between competitions were trivial to small in nature, with tier 1 competition associated with a larger number of RHIE bouts (6.5 ± 1.4 vs. 5.7 ± 1.5, effect size, *ES =* 0.55) and efforts per bout (3.0 ± 1.1 vs. 2.4 ± 1.2, *ES* = 0.52). Collisions comprised a greater proportion of total HIE for forwards within tier 1 competition compared to tier 2 competition. The hooker (mean difference: 4 [−10 to 14]; *ES =* 0.30, small), lock (mean difference: 5 [−12 to 23]; *ES* = 0.36, small) and backrow (mean difference: 8 [−10 to 15]; *ES* = 0.54, small) positions engaged in more collisions during tier 1 competition compared to tier 2 competition. These findings can be used by athletic performance staff to design game-specific drills and recovery strategies during different competition weeks to ensure players are appropriately prepared for the differing demands of elite rugby competition.

## 1. Introduction

Wearable microsensor technology (i.e., global positioning systems, accelerometers, gyroscopes and magnetometers) provide a practical means of quantifying the movement and collision demands of rugby union [1,2,3,4,5]. Typically, running, acceleration and collision events are described across the course of match-play [6,7]. However, recent studies have focused on “clusters” of high-intensity activity, commonly referred to as repeated high-intensity effort (RHIE) activity [8]. A RHIE bout is defined as ≥3 high-intensity efforts (running, acceleration or collisions) with ≤21 s recovery between efforts. Early work used video time motion analysis to understand RHIE in hockey [9], soccer [10] and rugby league match-play [11,12], highlighting that RHIE activity typically occurs during critical moments of play [13,14] (e.g., tries scored and conceded, shots on goal). Due to the time-consuming and labour-intensive nature of video analysis, these studies were limited to small sample sizes. The advent of wearable microsensor technology has assisted athletic performance staff in capturing a much greater number of matches, with some systems capable of automatically detecting RHIE bouts within match-play. Although single running, acceleration and collision efforts are well understood [5,15,16], there is a dearth of literature describing the positional and contextual factors that influence RHIE activity in elite rugby union match-play. 

RHIE activity is well established as a key performance indicator within the rugby codes [2,8,17]. Tierney and colleagues [18] observed that successful attacks (entries into opponent’s 22 m line) were positively associated with the number of RHIE bouts completed by players. Furthermore, the number of RHIE bouts completed by players is greater in winning teams [19,20] and those competing at a higher playing level [21]. Longer ball-in-play times also afford players greater opportunity for extended durations of RHIE bout activity, which in turn increases the physical cost on players [22]. The number of RHIE bouts performed during match-play has also been shown to be position-specific and with decreases observed from the first to second half [23]. Studies have also demonstrated that increasing the number of efforts within a RHIE bout negatively influences subsequent running activity and skill execution [24]. Finally, capturing match-play RHIE data allows coaches to understand the most extreme demands of match-play to make informed decisions on program design, facilitating physical preparation and recovery strategies. 

Despite the growing body of literature describing the physical demands of elite rugby union match-play, relatively little is known about the RHIE demands of different playing standards. Within the European rugby union, players are required to compete in two competitions, the European Rugby Cup Championship (tier 1) and a league-based format (tier 2). Given that these competitions occur concurrently within the rugby union season, providing an in-depth understanding of the potential increase in physical demands (e.g., RHIE) associated with tier 1 games would allow practitioners to make data-informed decisions regarding the game and position-specific content of training in preparation for these fixtures. Therefore, the purpose of this study was to quantify the physical demands of two elite European rugby union competitions, with special reference to RHIE activity. We hypothesised that the higher standard of competition would be associated with a greater frequency and intensity of RHIE activity. 

## 2. Methods

**Figure 1:**
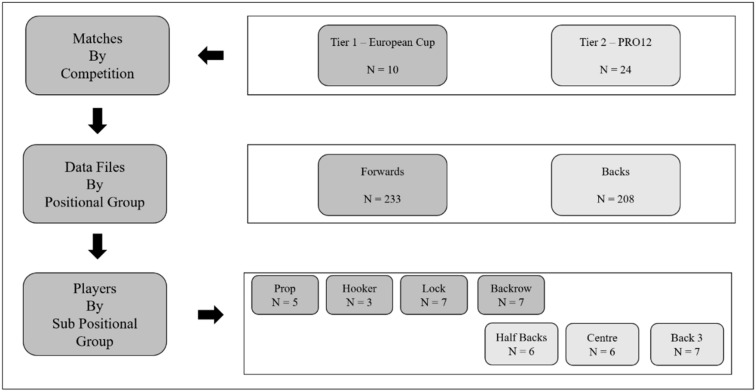
The experimental design of the current investigation details the specific breakdown of data files across competition games, positional groups and sub positional groups.

This observational study examined the movement demands and RHIE activity of two standards of elite rugby union match-play using GPS (and associated microsensor) technology. Forty-one (*n* = 41) elite rugby union players [(mean ± SD) age 26 ± 4 years; height 184.6 ± 6.7 cm; body mass 103.4 ± 6.7 kg)] with a professional full-time playing experience of 6.5 ± 2.5 years participated in the study. Players were classified as backs (*n* = 19) or forwards (*n* = 22), and further divided into eight specific positions of prop (*n* = 5), hooker (*n* = 3), second row (*n* = 7), backrow (*n* = 7), scrumhalf (*n* = 2), flyhalf (*n* = 4), centre (*n* = 6) and back three (*n* = 7). Before data collection, all subjects were informed of the risks and benefits of the study and gave informed consent. Ethical approval was granted by the Institutional Human Ethics Committee of the TU Dublin Research Ethics and Integrity Committee (REC-PG2R-201819). The observational period resulted in 441 individual game files being collected across the tier 1 (European Rugby Cup Championship, *n* = 8) and tier 2 (Pro12, *n* = 24) competitions (Figure 1). Players were required to complete a minimum of 55 min of game time for data to be included. All games took place between 13:00 and 20:00 h. Ambient temperature during match-play ranged from 7 °C to 21 °C. Prior to match-play, players were advised to maintain their normal diet with a specific focus on increased carbohydrate and fluid consumption. 

During matches, participants wore an individual GPS unit (S5, Catapult Innovations, Scoresby, VIC, Australia), sampling at 10 Hz. The GPS unit was encased within the players’ jerseys in a GPS pouch between the shoulder blades in the upper thoracic spine region. This ensured that the players’ range of movement in the upper limbs and torso was not restricted. The device was activated, and satellite lock was established for a minimum of 30 min before the commencement of each match [25]. This GPS technology has been shown to provide valid and reliable measurements of distance, low- and high-speed movements, speed and collisions [1,26,27,28,29,30]. Each individual player wore the same GPS unit for each match during this study to minimise inter-unit error [30,31]. Following each match, GPS data were downloaded using the same proprietary software (Catapult, OpenField Ver:1.12, Catapult Innovations, Scoresby, VIC, Australia). 

Across the investigation period, the horizontal dilution of precision (HDOP) was 0.8 ± 0.2, with an average satellite count of 10.6 ± 1.6. Each file was trimmed so that only active playing data were included for further analysis. The proprietary software provided instantaneous raw velocity data sampling at 10 Hz; summary data were synced to the manufacturer’s cloud platform “OpenField” and then exported into a customised Microsoft Excel spreadsheet (Microsoft, Redmond, WA, USA). The spreadsheet allowed the analysis of distance covered in the following categories: total distance, running distance (≥4.4 m∙s^−1^), high-speed running (≥5.5 m∙s^−1^), sprint distance (≥7 m∙s^−1^), accelerations (≥2 m∙s^−2^), decelerations (≤−2 m∙s^−2^) and collisions (≥4 g^−1^). Additional measures collected included peak velocity, total high-intensity efforts (HIE, ≥4.4 m∙s^−1^ with a dwell time of 0.4 s), acceleration efforts (≥2 m∙s^−2^), deceleration events (≤−2 m∙s^−2^) and collisions (impacts ≥4 g^−1^ force). Repeated high-intensity efforts (RHIE) [21] were also recorded. The software algorithm to capture RHIE events was set to ≥3 efforts within a 21 s time window. The total number of RHIE bouts, the maximal number of efforts within a RHIE bout and recovery times between RHIE bouts were also recorded. All statistical analyses were performed using SPSS for Windows (Version 22; SPSS Inc., Chicago, IL, USA). Descriptive analysis and assumptions of normality were verified before parametric statistical analysis was used. Using the Kolmogorov–Smirnov test, the normality of the data distribution was checked, and all dependent parameters were normally distributed (*p* > 0.05). The analysis was performed using a 2-way (position × competition) mixed design analysis of variance (ANOVA). When an interaction occurred, a Bonferroni post hoc correction was used to detect differences between competitions (2 levels: tier 1 and tier 2) and among positions (8 levels: prop, hooker, lock, backrow, scrumhalf, flyhalf, centre and back three). Standardised effect sizes (ES) were calculated as ≤0.20 = trivial, 0.21–0.60 = small, 0.61–1.20 = moderate, 1.21–2.00 = large and 2.01–4.00 = very large [31].

## 3. Results

### 3.1. Whole-Game Physical Demands of Tiers 1 and 2 Match-Play

**Figure 2:**
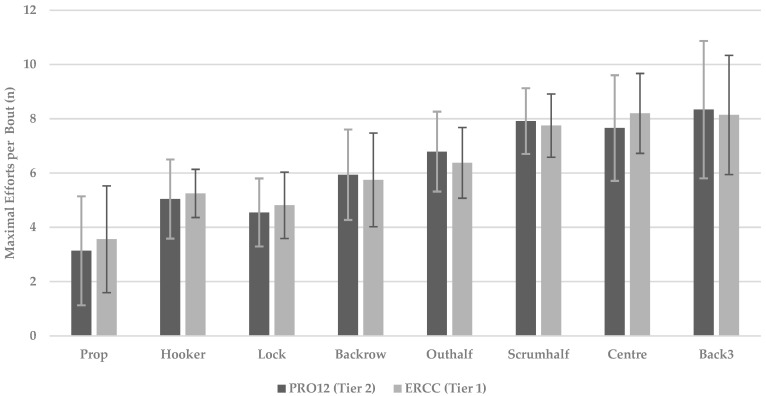
The maximal RHIE efforts per bout across competition with respect of sub-positional grouping. Data reported as mean ± SD.

The whole-game physical demands of tier 1 and tier 2 match-play are reported in Table 1 and Figure 2. *Small* differences were observed between competitions for total distance (mean difference: −363 [−212 to −657]; *p* = 0.391; *ES =* −0.29), distance covered running (mean difference: −113 (−83 to −243); *p* = 0.040; *ES* = −0.35) and total number of HIEs (mean difference: −18 (−4 to −33); *p* = 0.656; *ES* = −0.32), with higher activity profiles observed in tier 2 competition. Tier 1 competition was associated with a greater number of collisions (mean difference: 5 (2 to 17); *p* = 0.431; *ES =* 0.32; small), RHIE bouts (mean difference: 0.4 (1.1 to 2.1); *p* = 0.301; *ES* = 0.55; small) and average number of efforts per RHIE bout (mean difference: 0.6 (−1.2 to 2.0); *p* = 0.478; *ES =* 0.52; small). 

### 3.2. Running Demands of Tiers 1 and 2 Match-Play

The activity profiles for specific positions in tier 1 and tier 2 competitions are shown in Table 2. Differences in total distance were observed between tier 1 and tier 2 games; these were position-specific and ranged from trivial to moderate (*p* ≤ 0.05; *ES*: 0.10–0.52; *trivial–moderate*) in nature. Similar trends were reported for running and HSR, with positional differences observed within the data. The props (mean difference: −55 [−106 to 123]; *ES =* −0.58; *small*), lock (mean difference: −54 [−78 to 33]; *ES =* −0.24; *small*), backrow (mean difference: −57 [−108 to 99]; *ES =* −0.25; *small*), half back (mean difference: −151 [−343 to −77]; *ES =* −0.67; *moderate*) and back three (mean difference: −229 [−344 to −84]; *ES =* −1.05; *moderate*) positions all covered significantly greater running distance during tier 2 games than tier 1 games. 

### 3.3. Collision and RHIE Bout Frequency in Tiers 1 and 2 Match-Play

**Figure 3:**
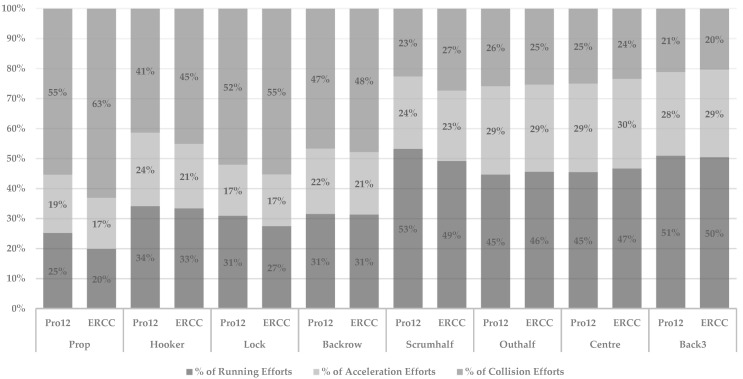
The percentage of High intensity effort distribution across competition with respect of sub-positional grouping. Data reported as relative percentage of total effort count.

The hooker (mean difference: 4 [−10 to 14]; *ES = 0.30*; *small*), lock (mean difference: 5 [−12 to 23]; *ES =* 0.36; *small*) and backrow (mean difference: 8 [−10 to 15]; *ES =* 0.54; *small*) positions engaged in a greater number of collisions during tier 1 competition than tier 2 competition. Conversely, in the back line, centres (mean difference: −7 [−16 to 12]; *ES =* −0.69; *moderate*) and the back three (mean difference: −7 [−12 to 23]; *ES =* −0.86; *moderate*) engaged in fewer collisions during tier 1 games. Finally, when RHIE bouts were analysed, the hooker (mean difference: 1.6 [0.3 to 3.5]; *ES =* 0.60; *moderate*) and lock (mean difference: 0.7 [−03 to 3.2]; *ES =* 0.24; *small*) positions engaged in more RHIE bouts during tier 1 than tier 2 matches. Finally, there was a noted change in the percentage distribution of efforts between competition across positions, which is shown in Figure 3; differences were trivial to moderate in nature depending on position (*p* ≤ 0.05; *ES*: 0.09–0.48; *trivial–moderate*).

## 4. Discussion

The current investigation aimed to understand the differences in RHIE activity between tier 1 and tier 2 rugby union competitions in the northern hemisphere. Furthermore, we aimed to understand the differences in positional locomotor demands across competitions. The main findings of the investigation were that within tier 1 competitions, players’ demands increased across collisions, RHIE bouts, efforts per RHIE and average efforts per bout compared to tier 2 competition. Furthermore, total distance, running and HSR demands were lower within tier 1 competition compared to tier 2, suggesting greater collision and repeated effort demands at higher levels of competition. In contrast to the above findings, similarities were shown across total high-intensity efforts completed by players in tier 1 and tier 2 competitions. Overall, the data suggest a trend toward lower running-based locomotor demands per running effort and higher collision and RHIE demands during tier 1 competition across all positions within northern hemisphere rugby unions. It is crucial that practitioners understand the differences in competition characteristics across locomotor and collision-based requirements, especially when considering how best to prepare players across a season. Coaches can aim to utilise these data to tailor their weekly training content to best reflect the competition their team is preparing for within their seasonal calendar structure.

Our data represent the first insight into position- and competition-based differences across rugby union locomotor profiles. The data show that there is a consistent reduction in running performance of rugby union players across competitions, with a significant reduction in total distance, running and HSR observed between tier 1 and tier 2 competitions. Furthermore, there was a noted increase in collision-based demands between competition standards, particularly within the abrasive forward positions of prop, hooker and lock. When analysed across positions, all positional groups were shown to have a reduction in running-based demands, with the exception of the prop and hooker positions, depending on the measures analysed. External training load measures such as total distance, running and HSR are commonly utilised by performance staff to develop training content that can increase players’ tolerance to these running demands within match situations. Head coaches and associate staff (forwards and backs coaches) can, however, sometimes become over-interested in players’ ability to accumulate running distance within matches without understanding the contextual nuances of competition. Our data show that the level of competition has an impact on players’ ability to accumulate running-based distances; this in conjunction with increased collision and RHIE demands may suggest that higher tier games from a physical perspective are determined by players’ ability to link efforts and the ability of players to win the “collision contest”, with running-based data being a secondary construct of match analysis. Our data contrast with the typical qualitative perceptive commentary that tier 1 competition is “more intense” than tier 2 match-play, given that this idea is not supported by the traditional locomotor metrics, namely total distance, running and HSR. Furthermore, it is plausible that less space to cover distance is available during tier 1 competition compared to tier 2 competition due to the higher skill level, defensive structures, tournament tactics and style of play of players and teams, which explains the reduced running requirements across competition levels. This may suggest that the analysis of running data alone offers value only from a preparatory perspective and that layering on effort and collision measures such as RHIE, efforts per bout and collisions may provide a more holistic understanding of the requirements of players during different competitions across a competitive season. 

The number of RHIE bouts performed during match-play has previously been shown to be position-specific, with a reduction in RHIE between halves of play [23]. Studies have also demonstrated that increasing the number of efforts within an RHIE bout can negatively influence subsequent locomotion and skill execution [24]. The current investigation observed that players’ total high-intensity efforts were similar irrespective of the tier of competition. However, while the total effort count was similar between tiers, the composition of bouts was shown to be different. Indeed, not only was there a noticeable increase in the average efforts per RHIE bout between competition (ES: 0.10; trivial), but maximal efforts per RHIE bout (ES: 0.55; small) were also shown to increase within tier 1 competition. Interestingly, from a positional perspective, the tight five groupings of prop, hooker and lock were identified as having greater maximal efforts per RHIE bout within tier 1 compared to tier 2. These data suggest that in higher tier competitions, the tight five will accumulate increased physical and physiological demands compared to lower tier competitions. Indeed, the RHIE bout creation regarding total effort output as measured by HIE also showed a greater rate of efforts being grouped together within positions during tier 1 games compared to tier 2 for forwards (3.7 vs. 3.6) and backs (9.9 vs. 9.7). This general trend of HIE input and RHIE bout creation was also seen within positions, with both forwards (4.8 ± 2.0 vs. 4.9 ± 1.8) and backs (7.8 ± 2.1 vs. 7.8 ± 1.8) having a greater average demand. Specifically, there is maximal within-bout effort RHIE demand in tier 1 compared to tier 2. From a coaching perspective, it is common for coaches to anecdotally suggest that players accumulate a greater number of phases during tier 1 competition compared to tier 2. This is often associated with an increase in opposition quality at both personal (players on field) and system (Game plans and coaching) levels [24]. The current study is the first of its kind to show positional differences in RHIE with respect to competition. Overall, the highlighted contextual factors associated with tier 1 games result in a greater number of phases being required to cross the “gain line” whilst also resulting in more contested and confrontational components to each play of the ball. These contextual factors may explain the data presented, given that the tight five (props, hookers and locks) all showcased a decrease in overall efforts with an increase in the proportion of contact efforts relative to the total when comparing across competition standards. Finally, of note from a coaching perspective was that even though an overall decrease in total effort across competitions with respect to total efforts was seen, an increase in the typical bout size and maximal efforts per bout was noticed when comparing tier 1 to tier 2. Coaches can now construct training drills that allow players to replicate the high-intensity effort and RHIE bout requirements depending on the match level the team is preparing for.

Collision demands were higher in tier 1 matches compared to tier 2 matches. This trend was highlighted specifically within the forward positions and more significantly within the abrasive forward positional groups of prop, hooker and lock. Interestingly, we also found a similar level of collision efforts as a relative contribution of overall efforts, with ~51% of a forward’s total effort accumulation coming from collisions compared to backs, who had ~23% of effort accumulation from collisions. Figure 2 highlights in detail the specific changes found in the effort composition across different positions depending on the level of competition. A clear trend towards an increase in the overall contribution of collisions was seen when comparing tier 1 and tier 2 within the forward positions. The higher collision characteristics across tier 1 competition levels are in line with previous literature within rugby unions [29,30]. These findings are of particular importance to practitioners, who must ensure that players have the appropriate physical qualities to dominate the collision area during match-play [3,4,27,30] whilst also ensuring that these players have the technical proficiency and running-related fitness to maintain the ability to link repeated efforts together across the full duration of match-play [27]. The highlighted increased collision demand may be reflective of the anecdotal review of elite coaches and players who often describe tier 1 competition as having an “increased level of intensity” compared to tier 2 competition. Furthermore, the noted increase in collisions may provide some insight into possible fatigue-induced running decrements seen within rugby unions [27,29,30], whereby the increased collision demands affect broader metrics such as total distance, running or HSR. Given the intermittent nature of the sport, this type of metabolic fatigue associated with the backs-dominant effort action of running-based demand allows for a sustained output of efforts across competition types, whilst the more collision-dominant demand within tier 1 competition may be related to the forwards’ position deterioration of HSR and RHIE bouts. These noted reductions may be attributed to the increased collision requirements of competition. Given that the measures are derived from inertial sensors, particularly collision-related measures, this may suggest to practitioners that these measures offer greater utility in differentiating the physical characteristics associated with different professional rugby union competition levels. However, practitioners must ensure that these measures are valid, reliable and sensitive to change within their own environments before reporting them to key stakeholders on a consistent basis.

Several limitations of this study warrant discussion. The capture method of participants was identified to allow comparison of “full” game demands when comparing across position. The comparative dataset is quite small and difficult to increase due to the knockout nature of cup competitions, yet these data are most relevant as they are representative of the highest opposition demands. Within this study, the use of absolute banding was employed for the assessment of running-based demands, and this fails to accurately account for the unique and different physiological make-up and physical capacity of each athlete within a position. A potential drawback of the current study design was the application of these absolute speed, acceleration and g-force bands when determining the positional running profile within the repeated effort framework. A potential recommendation for future studies would be the use of individualised speed, acceleration and g-force bandings per individual to assess the demands of the game relative to positional and individual profiles. However, a potential counter argument would be that absolute bands can identify top performers within a position based on absolute expression of work, including the development of positional norms that may need to be attained to manipulate field performance. Future research should aim to understand the periodisation of RHIE across positional lines and phases of the season to understand how coaching staff manipulate players’ exposure to these important events within a training context. Additionally, the data are representative of one team; as such, the team’s technical and tactical abilities and set-up for games potentially impact the dataset. Consequently, this study could be considered a case study with respect to competition differences in RHIE. Furthermore, the development of an assessment tool for RHIE accumulation is warranted to understand if a relationship between exposure to RHIE within training can impact players’ ability to accumulate these efforts within training and competition.

## 5. Conclusions

The design of training programmes that are reflective of the upcoming competition and previous loads are important to ensure optimal player performance and optimal recovery from previous competition demands. Therefore, the use of data in conjunction with coaching education will significantly impact the type of physical loads that players would be exposed to, and inversely, the recovery strategies implemented acutely between competition levels; this could be an important advantage for player health. The collaboration of practitioners between competition levels and teams seems to be of the utmost importance. Currently, performance teams face the challenge of concurrent competitive fixtures occurring within a single playing season during a northern hemisphere rugby union season. The current investigation sought to understand how the physical demands of rugby unions change with respect to the tier of competition. Our data show that RHIE demands were higher in tier 1 competition compared to tier 2 competition, highlighting that this measure may be an effective means by which to classify game demands across competitions. Specific changes in RHIE were shown across bout size in terms of efforts achieved and in the maximal efforts per bout. This is an important consideration for performance staff given the increase in typical bout size, and the maximal number of efforts within the largest bouts highlights the increased physical demand that elite opposition provides. Interestingly, it was seen that all running-based parameters such as total distance, running and HSR were lower in tier 1 competition compared to tier 2 competition. Further to this, we observed a greater collision demand specifically for the forward positional group during tier 1 competition. Coaches need to understand that at higher tiers of competition, it appears as if the collision contest is an important determinant of game intensity and physical demands. To conclude our data, we suggest that rugby union players require specific physical preparation for different levels of competition. Players may need specific preparation for the higher RHIE, bout and collision demands at higher levels of competition. 

## Figures and Tables

**Table 1:** The competition differences between League and European cup for running performance and RHIE variables within Rugby Union. Data reported as mean ± SD, Mean difference (95% CI) and Effect Size (95% CI).

Performance Variables	European Cup (Tier 1)	PRO12 (Tier 2)	Mean Difference (95% CI)	*p* Value	Effect Size (95% CI)
Total Distance (m)	5709 ± 1002	6051 ± 1301 *	362 (212 to 657)	*p* = 0.391	0.29 (0.04 to 0.43)
Running (m)	612 ± 347	705 ± 381 *	113 (83 to 243)	*p* = 0.040	0.35 (0.09 to 0.54)
High Speed Running (m)	226 ± 183	263 ± 210	57 (12 to 109)	*p* = 0.791	0.19 (0.05 to 0.31)
High Intensity Efforts (*n*)	153 ± 40	167 ± 48	18 (4 to 33)	*p* = 0.656	0.32 (0.11 to−0.64)
Collisions (*n*)	48 ± 15 *	43 ± 16	5 (2 to 17)	*p* = 0.431	0.32 (0.18 to 0.58)
RHIE Bouts (*n*)	6.5 ± 1.4 *	5.7 ± 1.5	0.4 (1.1 to 2.1)	*p* = 0.301	0.55 (0.28 to 0.72)
Efforts Per RHIE Bout (*n*)	3.0 ± 1.1 *	2.4 ± 1.2	0.6 (−1.2 to 2.0)	*p* = 0.478	0.52 (0.23 to 0.64)
Mean Efforts Per RHIE Bout (*n*)	4.0 ± 0.9	3.9 ± 1.0	0.3 (−1.3 to 2.1)	*p* = 0.675	0.10 (0.03 to 0.25)

Running (≥4.4 m∙s^−1^); high-speed running (≥5.5 m∙s^−1^) and collisions (≥4 g^−1^) and RHIE: Repeated High Intensity Efforts. * Significant difference between competition across analyzed variable; CI: Confidence interval.

**Table 2:** The running and RHIE variables with respect of position and competition within Rugby Union. Data reported as mean SD, Mean difference (95% CI) and Effect size (95% CI).

Performance Variables	Competition	Prop	Hooker	Lock	Backrow	Half Backs	Centre	Back 3
Total Distance (m)	PRO12 (Tier 2)	4613 ± 1249	5289 ± 1134 ^a^	5609 ± 1161 *^ab^	6319 ± 1134 *^abc^	6515 ± 1105 *	6537 ± 1048 *	6631 ± 881 *
	European Cup (Tier 1)	4716 ± 622	5314 ± 476 ^a^	5440 ± 993 ^a^	5403 ± 980 ^a^	6385 ± 507	6427 ± 985	6311 ± 516
	Diff 95% CI	201 (98 to 354)	45 (23 to 223)	−187 (123 to −343)	−976 (−433 to −1098)	−143 (−99 to −260)	−113 (−76 to −324)	−324 (−123 to −543)
	ES	0.10	0.02	−0.15	−1.04	−0.17	−0.11	−0.44
Running (m)	PRO12 (Tier 2)	206 ± 103 *	435 ± 137 ^a^	416 ± 170 *^a^	590 ± 211 *^abc^	1008 ± 250 *^e^	966 ± 242 *	1235 ± 255 *^de^
	European Cup (Tier 1)	155 ± 66	470 ± 121 *^ac^	379 ± 125	533 ± 243 ^abc^	857 ± 197	948 ± 250 ^d^	1006 ± 171 ^de^
	Diff 95% CI	−55 (−106 to 123)	43 (−23 to 98)	−54 (−78 to 33)	−57 (−108 to 99)	−151 (−343 to −77)	−18 (−56 to 33)	−229 (−344 to −84)
	ES	−0.58	0.27	−0.24	−0.25	−0.67	−0.07	−1.05
High Speed Running (m)	PRO12 (Tier 2)	42 ± 31	114 ± 52 ^ac^	80 ± 55 *^a^	160 ± 79 *^abc^	347 ± 109	398 ± 154 *	446 ± 114 *^de^
	European Cup (Tier 1)	32 ± 26	125 ± 44 *^ac^	68 ± 42 ^a^	156 ± 94 ^abc^	322 ± 105	357 ± 127	398 ± 98 ^de^
	Diff 95% CI	−10 (−23 to 12)	11 (−33 to 19)	−21 (−42 to 12)	−9 (−11 to 18)	−32 (−55 to −13)	−41 (−65 to −20)	−86 (−123 to −43)
	ES	−0.34	0.22	−0.24	−0.04	−0.23	−0.29	−0.54
High Intensity Efforts (*n*)	PRO12 (Tier 2)	99 ± 16	134 ± 35 ^a^	162 ± 40 ^ab^	177 ± 46 ^ab^	174 ± 40 *	185 ± 41 *^d^	193 ± 42 *^d^
	European Cup (Tier 1)	103 ± 27 *	150 ± 23 *^a^	173 ± 26 *^ab^	200 ± 36 *^abc^	163 ± 26	164 ± 25	182 ± 17 ^de^
	Diff 95% CI	5 (−21 to 33)	21 (11 to 43)	11 (−4 to 32)	31 (12 to 44)	−19 (−34 to −8)	−33 (−53 to −11)	−15 (−48 to − 8)
	ES	0.18	0.54	0.32	0.55	−0.32	−0.61	−0.35
Collisions (*n*)	PRO12 (Tier 2)	33 ± 13	40 ± 17 ^a^	51 ± 15 ^ab^	54 ± 12 ^ab^	34 ± 10	40 ± 13 *^df^	32 ± 9 *
	European Cup (Tier 1)	35 ± 7	44 ± 7 *^a^	56 ± 12 *^ab^	62 ± 17 *^abc^	33 ± 8	33 ± 6	25 ± 7
	Diff 95% CI	2 (−3 to 14)	4 (−10 to 14)	5 (−12 to 23)	8 (−10 to 15)	−1 (−18 to 14)	−7 (−16 to 12)	−7 (−12 to 23)
	ES	0.19	0.30	0.36	0.54	−0.11	−0.69	−0.86
RHIE Bouts (*n*)	PRO12 (Tier 2)	2.3 ± 1.7	5.9 ± 2.5 ^ac^	3.8 ± 1.5	8.6 ± 4.4 ^abc^	14.3 ± 4.2	17.8 ± 6.1 ^df^	16.8 ± 4.3 ^d^
	European Cup (Tier 1)	1.7 ± 1.3	7.5 ± 2.8 *^ac^	4.3 ± 2.5 *^a^	9.3 ± 4.6 *^abc^	13.3 ± 3.6	17.3 ± 5.5 ^df^	14.9 ± 3.2 ^d^
	Diff 95% CI	−0.7 (−1.2 to 0.9)	1.6 (0.3 to 3.5)	0.7 (−0.3 to 3.2)	1.2 (0.3 to 2.8)	−1.1 (−2.3 to −0.3)	−0.5 (−1.3 to 0.7)	−2.1 (−3.5 to −1.6)
	ES	−0.39	0.60	0.24	0.15	−0.25	−0.08	−0.50
Maximal Efforts Per RHIE Bout (*n*)	PRO12 (Tier 2)	3.1 ± 2.0	5.0 ± 1.5 ^a^	4.5 ± 1.3 ^a^	5.9 ± 1.7 ^abc^	7.4 ± 1.3	7.7 ± 1.9	7.9 ± 1.2 ^d^
	European Cup (Tier 1)	3.6 ± 2.0 *	5.3 ± 0.9 ^a^	4.8 ± 1.2 ^a^	6.1 ± 1.9 *^abc^	7.1 ± 1.2	8.2 ± 1.5 *^df^	7.8 ± 1.2 ^d^
	Diff 95% CI	0.5 (−0.8 to 1.3)	0.4 (−0.7 to 1.2)	0.3 (−0.5 to 1.5)	0.4 (−0.11 to 1.1)	0.3 (−0.6 to 1.7)	0.6 (−0.3 to 1.3)	0.3 (−0.5 to 0.5)
	ES	0.24	0.25	0.23	0.11	−0.23	0.29	−0.08

Diff: Difference; CI: Confidence interval; ES: Effect Size. Running (≥4.4 m∙s^−1^); high-speed running (≥5.5 m∙s^−1^) and collisions (≥4 g^−1^) and RHIE: Repeated High Intensity Efforts. * Difference between competition across variable of analysis (*p* ≤ 0.05). Significant difference (*p* ≤ 0.05) between position and; (a) prop; (b) hooker; (c) lock. Significant difference (*p* ≤ 0.05) between position and; (d) half backs; (e) centre; (f) back-three.

## Data Availability

The data presented in this study are available on reasonable request from the corresponding author.
